# Facilitators and barriers to implementation of integrated community case management of childhood illness: a qualitative case study of Kapiri Mposhi District

**DOI:** 10.1186/s12913-022-07867-w

**Published:** 2022-04-14

**Authors:** Johnson Vonje Riri, Adam Silumbwe, Chris Mweemba, Joseph Mumba Zulu

**Affiliations:** 1 Department of Health Policy and Management, School of Public Health, University of Zambia, P.O. Box 50110, Lusaka, Zambia; 2 Department of Epidemiology and Global Health, Umeå University, 901 87 Umeå, Sweden

**Keywords:** Barriers, Facilitators, Health system, Implementation, ICCM, Kapiri Mposhi, Zambia

## Abstract

**Background:**

Zambia adopted the Integrated Community Case Management (ICCM) of childhood illness strategy in May 2010, targeting populations in rural communities and hard-to-reach areas. However, evidence suggests that ICCM implementation in local health systems has been suboptimal. This study sought to explore facilitators and barriers to implementation of ICCM in the health system in Kapiri Mposhi District, Zambia.

**Methods:**

Data were gathered through 19 key informant interviews with district health managers, ICCM supervisors, health facility managers, and district health co-operating partners. The study was conducted in Kapiri Mposhi district, Zambia. Interviews were translated and transcribed verbatim. Data were were analyzed using thematic analysis in NVivo 11(QSR International).

**Results:**

Facilitators to implementation of ICCM consisted of community involvement and support for the program, active community case detection and timeliness of health services, the program was not considered a significant shift from other community-based health interventions, district leadership and ownership of the program, availability of national and district-level policies supporting ICCM and engagement of district co-operating partners. Program incompatibility with some socio-cultural and religious cotexts, stock-out of prerequisite drugs and supplies, staff reshuffle and redeployment, inadequate supervision of health facilities, and nonpayment of community health worker incentives inhibited implementation of ICCM.

**Conclusion:**

The study findings highlight key faciliators and barriers that should be considered by policy-makers, district health managers, ICCM supervisors, health facility managers, and co-operating partners, in designing context-specific strategies, to ensure successful implementation of ICCM in local health systems.

## Background

Integrated community case management (ICCM) of childhood illness is a strategy to increase access to effective case management of under-five marginalized children suffering from malaria, pneumonia, and diarrhoea in hard-to-reach areas who otherwise have limited or no access to life-saving treatments [1, 2]. It is a cost-effective strategy implemented by community health workers (CHWs) who are selected from their respective communities, trained in diagnosis and treatment of childhood illnesses and in identifying children in need of immediate referral [3–5]. The World Health Organization (WHO) and the United Nations Children’s Fund (UNICEF) recommend ICCM as a key public health strategy to increase coverage of high-quality life-saving treatment services for children, especially in malaria-endemic countries [4].

Since its introduction, ICCM has been widely adopted by many countries particularly in sub-Saharan Africa where there is documentation of its contribution to improved child health outcomes [4, 6]. However, evidence suggests that even with these positive child health outcomes, ICCM implementation in local health systems has not been optimal over the years [6–8]. Programatic experiences show that ICCM implementation is not easy, considering human and financial resource constraints, high levels of poverty, low literacy, poor infrastructure, weak health systems, and numerous other challenges facing healthcare systems in many countries where it’s been adopted [5, 9, 10].

A limited number of countries are implementing the ICCM strategy at scale and those that have scaled it up, struggle to maintain an acceptable level of service quality [5, 11, 12]. Most countries still rely on donor support to fund their ICCM programs, which often limits implementation coverage [3]. The ICCM activities are implemented only as and when there is funding for key activities during a given period. Furthermore, support for ICCM varies across different Ministry of Health (MoH) technical units depending on where the responsibility of a particular component of ICCM is assigned. For example, where malaria control programs are well-funded and distinct from ICCM, there are greater obstacles to implementation as malaria control programs provide little incentives to participate in ICCM program activities [5, 7].

Attaining the full benefits of ICCM requires that it be successfully implemented within the local health systems at all levels, with high-level of political commitment and broad-based community support to ensure sustainability [4]. Limited implementation of the ICCM program into health system inhibits the programs’ ability to achieve the intended goals and expected outcomes. Issues around coordination of ICCM activities within the MoH and other Ministries are particularly problematic, which also hampers implementation. While the MoH may sometimes engage other ministries, the Ministries of Finance are not always involved in ICCM policy discussions in many sub-Saharan African countries [7]. These challenges contribute to limited financing for ICCM program support in the national healthcare planning and resource allocation systems, which negatively impacts CHWs’ incentives as well as supply and demand elements, including quality of care [5, 13].

In May 2010, Zambia adopted the ICCM strategy and the implementation of ICCM in Zambia remains a challenge to date [3, 14]. Studies in Zambia have largely focused on the management of fever due to malaria and pneumonia [15], management of malaria using artemisinin-based combination therapy (ACT) and rapid diagnostic tests (RDT) [16], quality and safety of ICCM [17] and the relative cost-and-effectiveness of treating uncomplicated malaria [18]. Nonetheless, limited studies have explored the implementation of ICCM into the health system in Zambia. This study fills this gap by exploring facilitators and barriers to implementation of ICCM in the health system in Kapiri Mposhi District, Zambia.

## Materials and methods

### Zambian health system context

In 2018, the under-5 mortality rate in Zambia was 61 deaths per 1000 live births [19]. Zambia has wide geographical variations in under-five health indicators, with rural districts having the highest burden of under-five morbidity and mortality rates [3]. Distance to health facilities and shortage of health workers, especially in rural areas, continue to hinder accessibility to facility-based treatment for childhood illness [3]. Only 50% of the population in rural areas is within five kilometers of a health facility [20]. Zambia continues to experience shortages of health workers, skills-mix challenges and inequities in the distribution of health workers, which is skewed towards urban areas [21].

The ICCM package includes diagnosis and treatment of malaria, pneumonia, diarrhea, and screening and referral of malnutrition for under-five children [3, 14, 22]. We focused on malaria, pneumonia and diarrhea, diseases that cause the majority of under-five deaths. The initial design of ICCM was passive case detection, targeting under-five populations in rural communities and hard-to-reach areas [3]. Rural/hard-to-reach are areas with limited socioeconomic facilities characterized by limited/absence of infrastructure such as roads, health facilities, piped water and electricity[23]. In light of malaria elimination, active case detection has become part of ICCM [24, 25], taking both under-fives and adults, but we focused on the under-five. ICCM of childhood illness is one of the key malaria elimination interventions under case management [26].

The coordination and supervision of ICCM is at national, provincial and district levels [3, 14, 22]. ICCM is implemented by the Ministry of Health through mostly volunteer CHWs. In 2011, Zambia introduced a new cadre of paid CHWs, the Community Health Assistants (CHA), who provide treatment services at the health posts, including ICCM, and supervise volunteer CHWs [3, 14]. Conversely, CHWs are supervised by environmental health technicians (EHTs) in the study district.

### Study design

An exploratory qualitative case study design was employed because the implementation of ICCM into the health system has been under-researched, particularly in Zambia. The case study used an implementation research approach by involving key stakeholders responsible for child health and/or ICCM program design, planning, implementation, coordination, and monitoring &evaluation (M&E) in Kapiri Mposhi district.

### Study site

The study was conducted in the Kapiri Mposhi District, located in the Central Province of Zambia, about 185 km north of the capital city, Lusaka. The projected population for Kapiri Mposhi in 2019 was 297,484 people [27], with 53,189 being children under 5 years of age. The district has a total of 37 health facilities, with 2 hospitals, 23 health centres, and 12 health posts. By the third week of February 2020, all the 35 primary healthcare facilities support ICCM implementation in their catchment areas. In addition, the deaths due to malaria, pneumonia, and diarrhoea in the study district were 105, 59, and 47 per 1000 live births [28].

### Study population

The study population were district health managers, ICCM supervisors, health facility managers, and district health co-operating partners, involved in child health and/or ICCM program design, planning, implementation, coordination, and M&E from October 2018 to February 2020 and were available and willing to participate in the study.

### Sampling and recruitment of study participants

Purposive sampling was used to select study participants because it allows for the identification and selection of information-rich respondents related to the phenomenon of interest, and has been widely used in qualitative research [29]. To ensure adequate information on the barriers and facilitators to ICCM implementation in the health system, there is a need to target respondents with knowledge and experience in child health and/or ICCM program design, planning, implementation, coordination, and M&E. Purposive sampling, therefore, helped to identify information-rich district health managers, ICCM supervisors, health facility managers, and district health co-operating partners to explore barriers and facilitators to ICCM implementation in the district health system in Kapiri Mposhi district. Although purposive, participant recruitment was participatory by engaging the services of the District Health Officer (DHO) and district malaria focal point person, in recruiting study participants.

### Data collection

A total of 19 key informant interviews (KIIs) were conducted. Of these, 12 were male and seven were female respondents. Ten were district health managers, four ICCM supervisors, four health facility managers, and one district health co-operating partner (Table 1). The first author, who received postgraduate training in qualitative research conducted the interviews. Three experienced supervisors (AS, JMZ, and CM) in conducting various forms of qualitative research work and program evaluations provided supervision and support during data collection. The interviews were conducted in English, using an interview guide adapted from UNICEF’s case study on integrating childhood TB into maternal child health, HIV, and nutrition services [30]. It was pre-tested and further aligned before data collection. All interviews were audio-recorded.

**Table 1:** Key informant interviews

The participant categories	Sex		Number of interviews
	Male	Female	
District health managers	9	1	10
ICCM supervisors	2	2	4
Health facility managers	0	4	4
District health co-operating partners	1	0	1
**Total numbers of KIIs**	**12**	**7**	**19**

### Data management and analysis

The first author transcribed verbatim into Word documents all interview audios. The analysis started with familiarization of the data gained through reading and re-reading the transcripts and noting down initial ideas for analysis in an excel sheet. The thematic analysis approach “a method for identifying, analyzing, and reporting patterns (themes) within data,” was used [31]. Formatted transcribed were imported into NVivo 11 (QSR International) to aid storage, further organization, searching, and coding, to conduct an iterative analytical-qualitative analysis of transcripts. The coding process started by auto-sorting, then queried for word frequency and text search. The coded data were developed and shared with superviors (AS, JMZ, and CM) for their independent reviews. Once there was consensus on the coding structure and common views on the main themes and sub-themes, the code reports were generated. This was an iterative analytical process involving moving back and forth between data sources, codes, themes, and the study objectives. We defined barriers and facilitators as any factors that hindered or promoted ICCM implemention into the critical functions of health systems - governance, financing, planning, service delivery, M&E, and demand generation.

### Ethical considerations

Ethical clearances to conduct the study were obtained from the University of Zambia Biomedical Research Ethics Committee (UNZABREC) [IRB 00001131 of IORG 0000774, reference number 225-2019], while permission was sought from the National Health Research Authority (NHRA), Zambia. The Central Provincial Health Office (CPHO) and Kapiri Mposhi DHO provided additional authorization. Written informed consent was sought from all the participants before data collection. All procedures were performed in accordance with relevant guidelines.

## Results

This section presents the key findings from the analysis of barriers and facilitators to ICCM implementation in the district health system in Kapiri Mposhi district. Below is the final code-list that provided the basis for a structured data analysis (Table 2).

**Table 2:** Qualitative data analysis code-list

Main theme	Sub-theme
Facilitators to ICCM implementation in the district health system	1. Community-level factors • Community involvement and support • Active community case detection and timeliness of health services • Program not a significant shift from other community-based health interventions
	2. Leadership and stewardship • District leadership and ownership of the program • Availability of national and district-level documents supporting ICCM • Engagement of district health co-operating partners
Barriers to ICCM implementation in the district health system	3. Intervention related factors • Program incompatibility with socio-cultural and religious beliefs 4. Commodities and supply chain • Stock out of prerequisite drugs and supplies 5. Human resource for health management-related factors • Staff reshuffle and redeployment • Inadequate supervision of health facilities • Nonpayment of CHW incentives

### Facilitators to ICCM implementation in the district health system

#### Community-level factors

##### Community involvement and support for ICCM

The study participants indicated that CHWs, volunteers, political and traditional leaders, church representatives were involved in the ICCM program planning and implementation. These stakeholders participated in identifying and prioritizing comunity health needs, with the support and guidance of catchment health facilities. The church representatives, political and traditional leaders were stated to be the gateway to the communities and the health systems. Their acceptance and approval was an important factor to provide services to their subjects. This involvement not only ensured ICCM activity was included in the district-level action plan, but also encouraged community acceptance of the ICCM program.

*“Once you involved the head’s men, it’s very easy to convince the community because they live within the same premises with the headmen...”* [Participant_007; ICCM supervisor]

*“…during facility planning, facilities engage communities, volunteers, political leaders (Ward Councilors), and church representatives to highlight their needs which are then prioritized. If ICCM is identified as a priority, it would receive some funding allocation.”* [Participant_018; District health manager]

The community-based health structures, in particular, neighbourhood health committees (NHCs) and community health assistants (CHAs), positively facilitated ICCM implementation in the health system. These community structures played an important role in planning for ICCM and other health needs, including mobilization and selection of CHWs as well as building the trust and confidence of the communities in ICCM.

*“The participation from other community groups has been very good, you can talk of the NHCs, and these people are involved right from the start from the time a volunteer is chosen to go for the training under ICCM…”* [Participant_015; ICCM supervisor]

##### Active community case detection and timeliness of health services

Study participants indicated that the ICCM program involved active community malaria case detection by trained CHWs, also referred to as indexing of cases. They narrated that CHWs identifed positive malaria cases from their catchment health facilities, tracked and followed them to their respective households. They tested every member of the household and treated positive cases, and thereafter extended to test every other household member in a radius of 140 m. The process is repeated for another 140 m’ radius for every positive malaria case detected and treated, which ensured those that are asymptomatic but have the malaria parasite are detected and treated right at the community level and within households.

*“…the approach is an active way of managing cases or following up of patients unlike the ancient way of waiting for patients to come, so as a patient gets positive malaria, all the households members and the surrounding households are tested because of one case that has being reported at the clinic…”* [Participant_001; District health management]

The timeliness of health services within communities compared to the traditional health facilities, where one may end up spending the whole day with or without access to the much-needed treatment was valued by most respondents. Moreover, participants echoed that the community prefered easily accessible health services in terms of distance from their homes as well as those with acceptable and reasonable waiting times.

*“…I feel it will take a shorter time to manage these cases at a community level than at clinics because clients will not move long distances to get this service but the services will be on the doorsteps in their catchment area.”* [Participant_012; District health manager]

*“The communities also find it easier to quickly access medication because it's closer to where they are, closer to the people and it fits into one of our mission of the ministry, taking health services closer so it is a good intervention.”* [Participant_018; District health manager]

##### Program not a significant shift from other community-based health interventions

The perception of the ICCM as not being a significant shift from other community-based health interventions in Kapiri Mposhi district, positively fostered ICCM implementation. The study participants reported that the ICCM program comprised the common features that characterize the community-based health workforce, especially involving community structures in selecting CHWs. Other similar requirements stated are being a resident in the community where one will work and work voluntarily.

*“…we already had people that were trained in community health work, we used to call them community health workers, and they were trained to manage all the conditions in the community as well as provide health messages to the community”* [Participant_015; ICCM supervisor]

#### Leadership and stewardship of ICCM

##### District leadership and ownership of the program

The study participants indicated that district health leadership right from the ICCM program inception to the planning through to execution, monitoring, and controlling, has been a proactive. They provided the required information and participated in the early process of the roll-out, in particular, sensitized the traditional leadership, guided the selection, training, and supervised the deployment of CHWs. It was reported that such leadership traits made the partnership between district health managers and other partners organizations in ICCM implementation complementary. This positively facilitated ICCM implementation in the district health system.

*“…as long as you communicate the management team is ready to pick up and support in terms of for example some information, weekly you can get that information and we have also seen in terms of reporting, the timeliness and accuracy issues have improved, this is because of the district leadership is owning the process… we are seeing the support even here [at training hall], we have four pieces of training and in each of the training, there  is either one or two district managers, the district director was here [at training hall] and am sure he is still around but this is not the case in some districts, so that is one of the positive signs I have seen.”* [Participant_010; District health co-operating partner]

The existence of a district malaria elimination officer, positively fostered ICCM implementation, as he oversaw the coordination of ICCM activities in the district.

*“…we had a specific officer appointed to be in-charge of malaria who is now malaria elimination officer, so even that was a contributing factor there was no way we are going to leave this intervention aside when we have a designated officer with a funded position.”* [Participant_009; District health manager]

##### Availability of national and district-level documents supporting ICCM

Many respondents mentioned the national malaria strategic plan of 2017-2019, Malaria treatment guidelines, community health policy, RDT testing guidelines, NHCs guidelines, integrated management of childhood illnesses (IMCI), and the district action plans were critical for implementation of ICCM. These documents provided the necessary strategic program direction therefore facilitating ICCM implementation in the health system.

*“…the community health policy we have”* [Participant_014; Health facility manager]

*“…yes, for example, the ICCM has a manual that’s what we are using…”* [Participant_010; District health co-operating partner]

##### Engagement of district health co-operating partners

The study participants indicated that the engagement of district health co-operating partners especially the Churches Health Association of Zambia (CHAZ) and John Snow Inc. (JSI), positively fostered ICCM implementation. The CHAZ financed all ICCM training requirements of CHWs, district health managers, ICCM supervisors, and health facility managers, provided technical assistance and guidance in the initial stages of implementation, while JSI supported the transport and other logistical aspects. Participants narrated that without this support, the district was unable to implement ICCM.

*“Partners such as CHAZ have been supporting us in terms of training the community-based volunteers [CBVs], even in the going training. Then of course partners such as JSI Save and JSI Discover health projects equally come in to help in terms of transport and other logistics indeed.”* [Participant_017; District health manager]

*“…as a district or an institution on your own you may not manage it, so you need to lobe for support from co-operating partners such as CHAZ.”* [Participant_005; District health manager

### Barriers to implementation of ICCM in the district health system

#### Intervention related factors

##### Program incompatibility with socio-cultural and religious beliefs

The study participants reported that program incompatibilities with socio-cultural and religious beliefs negatively fostered ICCM implementation. The socio-cultural myths and misconceptions about blood withdrawal during active community malaria case findings presented cultural concerns to some communities, and this limited the acceptance of the ICCM program. The concerns were even more alarming when sampled blood was not used in the presence of the blood donor, family members, and/or community members, leaving room for them to associate blood withdrawal with *“satanic”* and/or *“ritual”* purposes.

*“You know, culturally, people become skeptical when it comes to issues that pertain to blood, but then the main issue is that if you get blood from someone and you take and go with it, then they remain thinking that this could be Satanist or I don't know what they [CHWs] will do with my blood and the like, even when you have explained something related to health.”* [Participant_002; District health manager]

Some religious beliefs were stated as a barrier to ICCM implementation, as they forbade their church members from accepting or using any form of healthcare service, including ICCM. The case, in particular, was the Zion religion who believed in divine healing, by emphasizing that their members pray to God or have their pastors pray for them whenever sick.

*“The community has accepted it [ICCM], unless those people, those who go to that church of the Zion don't go to the clinic, including ICCM; when they are sick, they go to their pastor to pray for them, they come to the clinic when they are in worse condition”* [Participant_014; Health facility manager]

#### Commodities and supply chain

##### Stock-out of prerequisite drugs and supplies

Persistent stock-outs of prerequisite ICCM drugs and supplies, especially amoxicillin for treating pneumonia, zinc sulfate tablets, and oral rehydration salts (ORS) for treating diarrhoea negatively affected implementation of the ICCM intervention, as community members had to travel long distances to the health facilities to access these treatments. Relatedly, the stock-outs of RDTs and gloves also affected the communities’ trust and confidence in the ICCM program and made implementation a challenge.

*“…when you look at the treatments for diarrhoea, pneumonia it’s not happening because the drugs are out of stock so, you find that when you talk of the community they are only treating malaria then this other illness they have to come here [health facility].”* [Participant_008; ICCM supervisor]

*“Yes like as of now there is no RDT, we don’t even know when it will come when they [CHWs] will start again going in the field or maybe it’s the end of them[CHWs] going in the community.”* [Participant_014; Health facility manager]

#### Human resource for health management-related factors

##### Staff reshuffle and redeployment

The respondents indicated that half of the initially trained district health managers during ICCM program roll-out in Kapiri Mposhi district was reshuffled and redeployed to other districts by the MoH. The few that remained have other duty assignments, apart from the malaria elimination officer, who also doubles as the ICCM focal point person. This negatively fostered ICCM implementation, as district managers’ support of the ICCM program was reportedly limited.

*“…we are very few despite having trained those numbers like 8, some of them are not specifically or directly related to malaria expect the malaria elimination officer, so they have other duties to do as well, so it becomes difficult to specifically focus on ICCM alone...”* [Participant_009; District health manager]

##### Inadequate supervision of health facilities

Inadequate supervision of health facilities by district health managers was reported to have negatively affected ICCM implementation. The study participants noted the lack of fuel, mechanically damaged motorcycles and/or vehicles, and financial constraints as the main impediment to the inadequate supervision of health facilities and CHWs. This is because the district personel had to travel to multiple health facilities.

*“…monitoring part, we have not adequately covered that one because we also have logistical challenges, there are times we don't have fuel, our vehicles break down, and we haven't given fuel to the facilities to enable staff to check what the community-based volunteers [CBVs] are doing, so monitoring has not been very adequate…*[Participant_005; District health management]

*“…we have a challenge of fuel because my only access to these places is a motorbike, so if fuel is available because the zones are apart, normally I do visit them[CHWs] once a month…”* [Participant_008; ICCM supervisor

##### Nonpayment of CHW incentives

Nonpayment of CHWs incentives, monetary and nonmonetary was indicated to be a barrier to ICCM implementation. This is because without incentivizing the CHWs who are key players in ICCM it is difficult to implement the program. The participants indicated that CHWs are entitled to some form of monetary remuneration tied to their performance; however, this entitlement remained an expectation and aspiration to many CHWs which affected their motivation and retention.

*“…in terms of financial support, there are entitled to some numeration based on their performance yes as they do their work there are some incentives that should be given to them…* [Participant_018; District health manager]

*“…we had around 3-4 groupings that were trained but they were not that active, I think there were those issues of incentives and the like after the training, there were not much going on…. lack of incentives to community volunteers, some may drop out few months after the training.”* [Participant_011; District health manager]

Others reported that nonmonetary incentives, like bicycles, raincoats, gumboots, and umbrellas were not provided although they were promised. The CHWs continued to walk on foot to their respective communities during community case detection. This was demotivating as some communities had vast geographical areas of land.

*“They are not yet given bicycles for them [CHWs] to move around, though they have been promised, I think we have not yet supported with any stationary to help out, basically that would also negatively affect the program.”* [Participant_002; District health manager]

*“…like this time is rain season, we needed the CHWs to give them bicycles for mobility and then also raincoats so that they are protected from rains and so on, all these things are not available.”* [Participant_003; ICCM supervisor]

The lack of identity cards or uniforms for CHWs was equally indicated as a barrier to ICCM implementation. This is because CHWs could not be accepted and recognized as health workers from the local facilities during active community case findings without the identity cards. This reportedly demotivated the CHWs. CHWs enjoyed various social benefits of working in the ICCM program, including the feeling of acomplishement seeing ill children recoverig from the common illnesses.

*“…one other challenge that we faced is the fact that these people [CHWs] are going in our communities without identification cards has been one issue that we have observed, so it's like they [community] cannot identify them [CHWs] in that sense or regard them [CHWs] as working under the MOH in that case.”* [Participant_017; District health manager]

## Discussion

Facilitators to implementation of ICCM consisted of community involvement and support for the program, active community case detection and timeliness of health services, the program not being considered a significant shift from other community-based health interventions, district leadership and ownership of the program, availability of national and district-level policies supporting ICCM and engagement of international co-operating partners. Program incompatibility with some socio-cultural and religious contexts, persistant stock-out of prerequisite drugs and supplies, staff reshuffle and redeployment, inadequate supervision of health facilities, and nonpayment of community health worker incentives inhibited implementation of ICCM in the health system.

Our study findings revealed that community involvement and support from key actors facilitate implementation of the ICCM program. This is consistent with a multi-country study in South Sudan, Uganda, and Zambia [32], Uganda [33, 34], and Nicaragua [35]. Community involvement and support ensured that the ICCM activity of training CHWs was included in the district action plans, facilitated the recognition and acceptance of ICCM, motivated the performance of CHWs and encouraged a sense of ownership among communities. These findings imply that the acceptance and motivation of CHWs in areas where ICCM is implemented is critical, and should not be overlooked to in efforts enhance implementation ourtcomes, as CHWs are the key drivers of the program.

The active community case detection and timeliness of health services fostered ICCM implementation through shortening the infectious period of patients by ensuring early diagnosis, treatment, and/or referrals, which is consistent with a study done in Sri Lanka [36]. This finding suggests that countries like Zambia seeking to eliminate malaria could strengthen active community case detection of asymptomatic cases through RDTs.

Strong ownership of the ICCM program by the district leadership facilitates ICCM implementation. This finding is supported by a study from the Democratic Republic of Congo (DRC) which showed that weak ownership of the program negatively affects the implementation of ICCM [37]. While ICCM stakeholders’ technical working group is in place at the national level to facilitate implementation, only a focal point person exists at the district level, which affects program coordination. A study by Bennett et al. [7] in six sub-Saharan African countries reported issues around coordination within the MoH and between ministries, which affected the implementation of ICCM. Our findings support the view that implementation can happen differently at the various levels of the health systems depending on the prevailing governance arrangements and supportive systems [38, 39].

Studies from Senegal [40] and the DRC [37] documented that the presence of national policy and implementation documents at the intermediate and operational levels are important factors for successful ICCM implementation. These findings agree with our study that national and district documents for ICCM are critical to providing strategic program direction. The presence of policies, regulations, and strategies at these levels promotes good leadership and governance for ICCM program implementation [41].

We found that the district health co-operating partners such as CHAZ financed ICCM training, provided technical guidance and direction during the initial phases of the program. The engagement of development partners such as UNICEF and USAID to provide financial resources and/or technical assistance has also been noted in other studies [40]. However, the intermittent funding of ICCM negatively affects the implementation of key activities, especially where partner programs are no longer supporting district ICCM activities. Further, non-payment of incentives affect motivation retention of CHWs and slows the rate of ICCM implementation, which is consistent with other previous studies [34, 42–44]. Therefore, to ensure sustained and effective ICCM implementation, consistent district health system financing must be enhanced [42].

Program incompatibility with the socio-cultural and religious beliefs limited implementation of ICCM. The socio-cultural myths and misconceptions about blood withdrawal are consistent with a similar multicountry study [32]. Religious beliefs, of forbidding church members, have also been reported in Malawi [45, 46]. The lack of user acceptance has been reported as a barrier to implementation [47]. These findings highlight the need to understand the compatibility of ICCM with the community in which it’s implemented. The socio-cultural and religious settings with associated barriers to adoption are critical to consider in designing context-specific implementation strategies.

Persistent stock-out of prerequisite ICCM drugs and supplies affects demand and threatens communities’ trust and confidence in the program. Gaps in ICCM commodities and supplies have been reported in a previous studies [32, 34, 42]. Inadequate logistics have the potential to make a good intervention, such as ICCM to be misconstrued as none performing, as it interrupts service delivery in the communities and affects the continuity of care, which hinders implementation [39]. As CHWs are the first level of contact with the healthcare system, stock-outs of essential medicines for treating these common childhood illnesses may lead to delayed access to care thereby increasing child mortality.

Assessing the facilitators and barriers is critical to ensuring successful implementation of the ICCM program. Various implementation research tools provide a framework for making these contextual assessments. For example, the consolidated framework for implementation research has been used to support systematic, comprehensive and timely understanding of facilitators and barriers. Further, implementation research frameworks can be useful for implementation teams to identify where adjustments and refinements to ICCM could be made to enhance program performance.

### Study strengths and limitations

The collection of data from various sources including district health managers, ICCM supervisors, health facility managers and district health co-operating partners ensured that broader perspectives were gathered which strengthened data triangulation. The qualitative team comprised a student and three supervisors with experience in conducting various forms of qualitative research work and program evaluations. The study had its limitations. Firstly, it was conducted in one district with unique context-specific attributes, a small sample of participants, and using one qualitative method which limits the transferability of study findings. However, providing a rich description of background data and the phenomena of interest led to an in-depth account of facilitators and barriers to ICCM implementation in the health system. Secondly, by the time of this study, half of the initially trained district managers in ICCM had left Kapiri Mposhi district, which probably did not capture all the relevant perspectives. Despite these shortcomings, the study provided a valuable contribution to the body of knowledge on facilitators and barriers to ICCM implementation in similar health systems contexts.

## Conclusion

The study findings suggest differences in ICCM implementation between the national and district level, possibly due to prevailing governance arrangements and supportive systems, which promoted or hindered ICCM implementation in the health system in Kapiri Mposhi. The key faciliators and barriers highlighted should be considered by policy-makers, district health managers, ICCM supervisors, health facility managers and district health co-operating partners, in designing context-specific implementation strategies to ensure the effectiveness of ICCM. Further, ICCM implementation teams must systematically assess potential facilitators and barriers in preparation for implementing of ICCM. This approach may help enhance implementation effectiveness of the ICCM program as well as identify improvements to implementation strategies for future implementation efforts.

## Data Availability

The data is publicly available and can be accessed from the first author (JRV) upon approval by the Zambia National Health Research Authority.

## Abbreviations

TB: Tuberculosis
HIV: Human Immunodeficiency Virus
IRB: Institutional Review Board
NGO: Non-Governmental Organization
USAID: United States Agency for International Development
